# Formation of Ethyl Carbamate during the Production Process of Cantonese Soy Sauce

**DOI:** 10.3390/molecules24081474

**Published:** 2019-04-15

**Authors:** Kai Zhou, Lorenzo Siroli, Francesca Patrignani, Yuanming Sun, Rosalba Lanciotti, Zhenlin Xu

**Affiliations:** 1 Guangdong Provincial Key Laboratory of Food Quality and Safety, College of Food Science, South China Agricultural University, Guangzhou 510642, China; zkjy1990@163.com (K.Z.); ymsun@scau.edu.cn (Y.S.); 2 Department of Agricultural and Food Sciences, Alma Mater Studiorum, University of Bologna, 47521 Cesena, Italy; lorenzo.siroli2@unibo.it (L.S.); francesca.patrignani@unibo.it (F.P.); rosalba.lanciotti@unibo.it (R.L.)

**Keywords:** ethyl carbamate, soy sauce, precursor, ethanol, accumulation stage

## Abstract

The aim of this work was to clarify the formation of ethyl carbamate (EC) and its influence factors throughout the production process of Cantonese soy sauce. The results showed that EC was not detected in the *koji*-making and early *moromi* fermentation stages, but started to be generated when pH of the *moromi* decreased to about 4.9—at the same time, the levels of ethanol, urea and citrulline increased significantly. Most EC was formed during raw soy sauce hot extraction (40.6%) and sterilization (42.9%) stages. The EC content exhibited the highest correlation with ethanol throughout the whole production process (R = 0.97). The simulation soy sauce produced in laboratory led the same conclusion—moreover, the contents of EC, ethanol and citrulline were higher in soy sauce fermented at 30 °C than in soy sauce fermented at 15 °C. Extraction of raw soy sauce by squeezing contributed little to EC formation. Further research showed that citrulline and ethanol led to significant increases in EC levels in raw soy sauce upon heating. These results indicate that ethanol and citrulline are two critical precursors of EC and that EC is mainly formed during the heat treatment stage of soy sauce.

## 1. Introduction

Soy sauce, an ancient condiment used for thousands of years, has long been one of the most popular seasonings in Asia. Currently, its popularity in the Western world is growing dramatically due to its characteristic aroma and bioactive components [1]. However, this popular condiment also tends to be contaminated by some harmful substances. Ethyl carbamate (EC), a byproduct that naturally forms in fermented foods, can cause tumors [2] and cell death [3]. This dangerous toxin was classified as a probable human carcinogen (Group 2A) by the International Agency for Research on Cancer (IARC) in 2007. Wu et al. found that soy sauce from Zhejiang Province contained 8–108 μg/L of EC, with an average level of 47 μg/L [4]. Koh and Kwon reported that the maximum EC content in Japanese-style soy sauce reached 128.9 μg/L [5]. Soy sauce has become the most remarkable source of EC exposure in some Asian countries due to its high consumption, especially among children [6,7]. Therefore, the presence of EC in soy sauce should be controlled to the lowest level with a feasible technical approach, and this control hinges on a full understanding of the formation of EC.

EC is naturally formed through the alcoholysis reaction between ethanol and carbamyl compounds [8,9]. The major precursors and dominantly emerging stages of EC differ with disparate food types and processing crafts. For instance, urea, the most important EC precursor in Chinese yellow wine, mainly accumulates during yeast metabolism [10], and cyanide is the precursor to EC in any form of cyanogenic plant material used in food production, such as Prunus species and sugar cane species [11,12]. Little research has been conducted on the major precursors of EC in the production of soy sauce because of the complexity and diversity of the soy sauce production process. In China, soy sauce is classified into high-salt liquid-state fermentation soy sauce (HLFSS) and low-salt solid-state fermentation soy sauce (LSFSS) according to fermentation craft [13]. Our group has previously proven that HLFSS is contaminated by higher level of EC than LSFSS [14]. *Koji*-making (the cooked soy beans and roasted wheat were inoculated with starter mold), *moromi* fermentation (the *koji* was mixed with salt brine), raw soy sauce extraction, and sterilization are four common processes of HLFSS production [15,16]. To the best of our knowledge, no data are available regarding changes in the levels of EC and its potential precursors during those soy sauce production processes.

Cantonese soy sauce, a kind of HLFSS indigenous to southern China, holds nearly half the share of the Chinese market for its unique flavor. The *moromi* fermentation usually lasts for almost 6 months and is divided into three stages: lactic acid fermentation, alcohol fermentation and an aging stage [17]. Ethanol is added before the raw soy sauce extraction—alternatively, sufficient captive yeast is supplied at the end of the lactic acid fermentation [18]. Our previous study found that ethanol showed a positive correlation with EC in different soy sauce products [14]. In some Japanese-style fermented soy sauce, the general ethanol content was 2%, and EC contents were relatively higher than other soy sauce [5]. However, the ethanol contents of Cantonese soy sauce were much lower than that of Japanese-style fermented soy sauce because no captive yeast is supplied during *moromi* fermentation, and the effect of low levels of ethanol on EC formation during the whole soy sauce production processes is still unknown. To achieve a high production efficiency with low production cost, some manufactories choose to increase fermentation temperature to shorten the soy sauce fermentation time. However, this increase in temperature not only increased the level of EC [12,19] but also affected the accumulation of EC precursors via microbial metabolism. The aim of this study is to determine the content of EC and its precursors during the whole production process of Cantonese soy sauce, in an effort to elucidate the critical EC accumulation stages and main precursors. Moreover, the impact of fermentation temperature on EC precursor accumulation was also investigated via two simulated fermented soy sauces under different fermentation temperatures. 

## 2. Results

### 2.1. Changes in EC, Ethanol and Urea in Cantonese Soy Sauce

**Figure 1:**
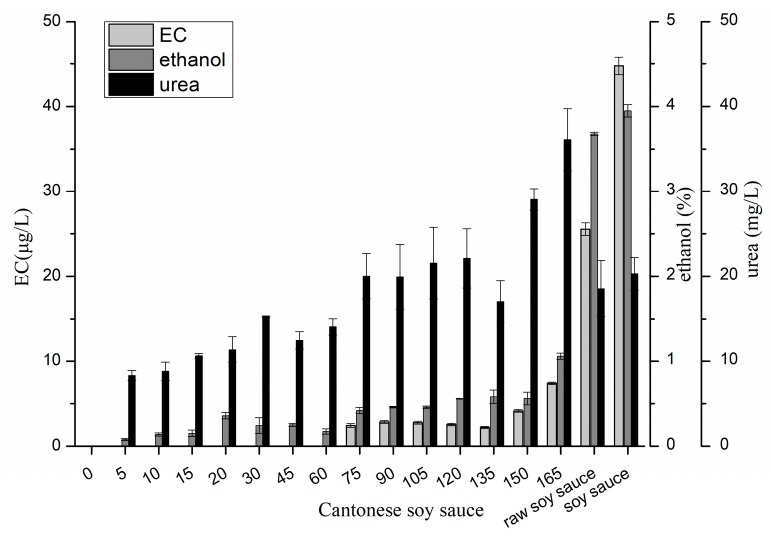
Changes of ethyl carbamate, ethanol and urea contents in Cantonese soy sauce (“0” on the X axis means the end of *koji*-making, similarly hereinafter).

Figure 1 shows the changes of EC content, ethanol content and urea content in the production processes of Cantonese soy sauce, which were in the range of non-detectable to 44.8 μg/kg, non-detectable to 3.9%, and non-detectable to 36.1 mg/L, respectively. During the fermentation stage, EC was not detected in *koji*-making and initial *moromi* fermentation stages, and not first detected until fermentation day 75. The EC content increased gradually between days 75 and 135, and then significantly increased to 7.4 μg/L at day 165. Compared with that in the *moromi*, the EC content in raw soy sauce and in soy sauce product significantly increased 3.5-fold and 6.1-fold, respectively. The ethanol and urea in the *moromi* were first detected at day five. When the EC was first detected, the ethanol and urea contents significantly increased to 0.4% and 20.0 mg/L, respectively. Additionally, the ethanol and urea contents did not remarkably increase during fermentation days 75–135. Both of them were significantly increased at day 165, similar to EC content. Although the ethanol content was under 1% during fermentation, it increased almost 3 times in soy sauce product because extra alcohol was added into the raw soy sauce for flavor enhancement. However, urea content decreased significantly in raw soy sauce. 

### 2.2. Changes of EC, Ethanol and Urea in Soy Sauce Produced in Laboratory

**Figure 2:**
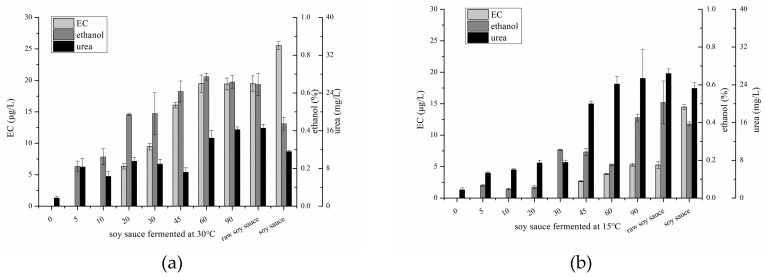
Changes of ethyl carbamate, ethanol and urea contents in soy sauce fermented at 30 °C (**a**) and 15 °C (**b**), respectively.

Except for fermentation temperature in the first month, the production processes of the two soy sauces fermented in the laboratory were the same. As shown in Figure 2, the EC content of soy sauce product fermented at 30 °C was 2.1-fold higher than in soy sauce product fermented at 15 °C, directly indicating that a high fermentation temperature resulted in a high EC level. Notably, EC in *moromi* fermented at 30 °C was not detected until day 20, and then significantly increased during day 20–60. In comparison, EC in *moromi* fermented at 15 °C was first detected on day 45, when the initial fermentation stage ended, moreover, the increase of EC in *moromi* fermented at 30°C was lower than that fermented at 15 °C. The formation of EC from squeezing process in raw soy sauce was almost negligible, but EC content significantly increased after sterilization at 85 °C for 30 min. The significant increase of ethanol and urea in *moromi* appeared earlier than the time when EC was first detected in both soy sauces. Ethanol is mainly produced by halophilic yeast (such as *Zygosaccharomyces rouxii*) when lactic acid fermentation is finished [16]. Its levels in *moromi* fermented at 30 °C were relatively higher than those in *moromi* fermented at 15 °C, moreover, the day when ethanol significantly increased to approximately 0.2% was earlier in *moromi* fermented at 30 °C than in soy sauce fermented at 15 °C. These results suggested that the lactic acid fermentation stage was shortened and that alcoholic fermentation was enhanced under high fermentation temperatures (as also demonstrated by pH values, Table 1), resulting in a high alcohol content. Urea had a trend to increase with prolonged fermentation time, and its levels in *moromi* mash fermented at 30 °C were relatively lower than those in *moromi* fermented at 15 °C after day 45. Urea and ethanol contents decreased after sterilization.

### 2.3. Metal Content, Cyanide, Free Amino Acids and pH

The metal contents are shown in Table S1. Metal content (Fe, Ca, Mg and Cu) in all samples hardly changed after 30 days of fermentation, and there was no significant correlation between each metal content and EC content in all kinds of soy sauce during the whole production processes. Cyanide content in Cantonese soy sauce decreased first and subsequently increased, while in two soy sauces fermented in the laboratory, it increased in the initial fermentation stage and then decreased (Figure S2). Most of the free amino acids increased during the fermentation stage. As shown in Table S2, the free amino acid of Cantonese soy sauce significantly increased during the first 60 days, and then increased a little in the later fermentation. The free amino acid contents in *moromi* fermented at 30 °C was a little higher than those fermented at 15 °C. The citrulline content increased with prolonged fermentation time (Table 2), moreover, the citrulline contents were higher in soy sauce fermented at 30 °C than in soy sauce fermented at 15 °C. Arginine increased at first but then decreased during the later fermentation stage. The pH of the three samples tended to decrease, and the higher the temperature was, the faster the pH dropped (Table 1). 

### 2.4. Correlation between Ethyl Carbamate and Its Precursors

Pearson correlations among EC, its potential precursors, and pH during soy sauce production processes (including *koji*-making, *moromi* fermentation, raw soy sauce extraction and sterilization) were conducted to understand the association between these influencing factors and EC content (Table 3). The EC level exhibited significant and positive correlations with ethanol in all soy sauce samples, especially in Cantonese soy sauce (R = 0.97). Similar results were found in soy sauce product [14] and in Chinese liquor [20]. This finding suggested that EC had the closest relationship with ethanol content among the possible precursors. In each independent sample, the pH showed significant correlations with EC, ethanol and citrulline. Urea showed a significant correlation with EC in soy sauce fermented in the laboratory, but an insignificant correlation coefficient between EC and urea was observed in Cantonese soy sauce (R = 0.26). 

### 2.5. EC Formation with Ethanol, Urea, Citrulline Addition and pH Adjustment

**Figure 3:**
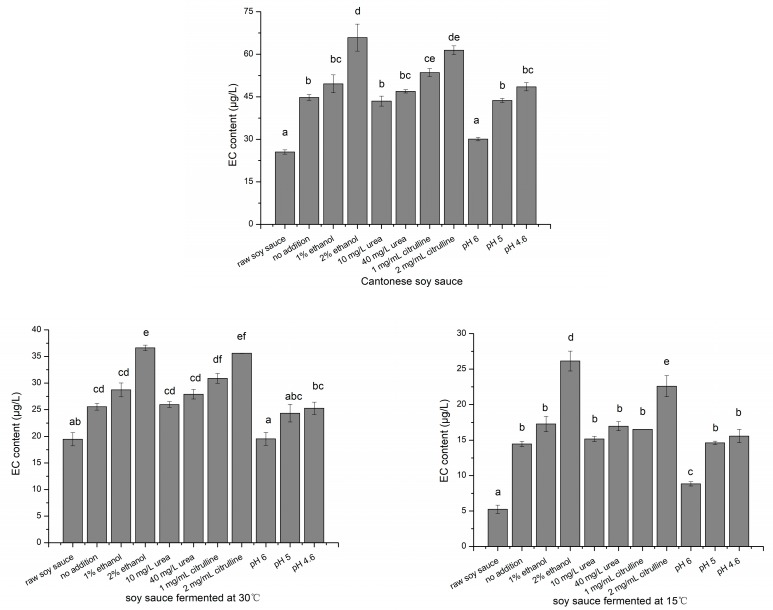
Ethyl carbamate (EC) content of raw soy sauce after heat treatment with possible EC precursors addition and different pH values (bars with different letters are significantly different from the control group (*p* < 0.05)).

EC was proven to form chemically from some EC precursors by heating (hot extraction stage and sterilization stage), and ethanol was one of the most critical EC precursors in soy sauce. To investigate the EC formation of the main carbamyl compound (urea or citrulline) during heat treatment process, ethanol, urea and citrulline, whose contents were relatively similar to those in raw soy sauce, were added to raw soy sauce. Then, the mixtures were heated at 85 °C for 30 min to simulate soy sauce sterilization. As shown in Figure 3, the EC level significantly increased after heat treatment. Under the original pH condition, urea (20 mg/L and 40 mg/L) showed no significant effect on the formation of EC in any soy sauce sample. In comparison, 1 mg/mL citrulline added to Cantonese raw soy sauce and 2 mg/mL citrulline added to all samples led to significant increases in EC. Similarly, Matsudo and Zhang [21,22] found that citrulline has a stronger ability than urea to generate EC in harsh pasteurization and simulation experiments. EC increased slightly after heating when the pH value of raw soy sauce was 6.0, and no significant differences in EC content were noted when the pH value of raw soy sauce was 4.6–5.0.

## 3. Discussion

### 3.1. Precursors of EC in Soy Sauce

EC is naturally formed in fermented food via nonenzymatic reactions of EC precursors, and its formation significantly depends on the food production process [23]. This study determined the main EC precursors and EC accumulation stages in Cantonese soy sauce throughout its production processes. Ethanol and citrulline in soy sauce were confirmed to be the two of most important contributions to EC, especially ethanol. In fact, the ethanol content showed the positive and significant correlation with EC content in all soy sauces (Table 3), and the addition of 2% ethanol resulted in a significant increase of EC after heat treatment (Figure 3). During the wine-making process, the production of EC is stimulated at the start of fermentation, and, in the presence of abundant alcohol [10], the excess ethanol resulted in the researcher neglecting its contribution to EC formation. In soy sauce, however, only approximately 2% ethanol was produced by halophilic yeast or artificial alcohol added to enhance soy sauce flavor [16]. The *moromi* contained low level of ethanol, even less than 1%. Consequently, ethanol became the limiting factor of EC formation. Moreover, the ethanol-enhanced solubility of free fatty acids in *moromi* mash stimulated the accumulation of citrulline during alcoholic fermentation [17]. In this study, the ethanol content correlated with the citrulline and urea levels in soy sauce produced in the laboratory (Table 3). Therefore, compared with the control of other precursors [24], the control of ethanol content with methods under the precondition of not harming soy sauce flavor was more effective in reducing EC content in soy sauce. 

The accumulation of citrulline was attributed to the catabolism of arginine by lactic acid bacteria (especially *Weissella confusa* and *Pediococcus acidilactici*) via the ADI pathway [22]. There are considerable amounts of arginine present at *moromi* fermentation (Table 2), thereby maintaining citrulline at a high level. However, this part of citrulline hardly transfers into EC during the fermentation stage due to the low temperature and high pH, which might be the reason for the weak correlation with EC (Table 3). Matsudo proved that addition of 50 mg/L urea affected lower than 5 μg/L formation of EC, but the urea in soy sauce from markets was less than 10 mg/L [21]. Although the urea level in Cantonese soy sauce product was 20.3 mg/L, which was higher than that of Japanese soy sauce and some alcoholic beverages, the addition of 40 mg/L urea showed no significant effect on the formation of EC in any of the soy sauce samples (Figure 3), while the addition of citrulline significantly increased the EC level upon heating. Therefore, citrulline was an EC precursor instead of urea during heat treatment of soy sauce. Yeast extract, which contains large amount of amino acids, is commonly added to soy sauce for strengthening the mellow fragrance in the production of Cantonese soy sauce. As citrulline and ethanol were not present in yeast extract, yeast extract was not responsible for EC formation. 

### 3.2. Critical Accumulation Stages of EC and Its Influencing Factors

The *koji*-making and *moromi* fermentation are two key stages for flavor characteristics formation of soy sauce. EC was not formed in the *koji*-making stage. Although EC precursors largely accumulated during the fermentation stage, the EC content only slightly increased because the reaction rate of EC formation was very low [11,21]. In Cantonese soy sauce, 83.5% of EC formed during the hot extraction of raw soy sauce and sterilization stage, indicating that these are the crucial stages of EC formation in soy sauce. The temperature of these stages was much higher than that of the fermentation stage [16]. Notably, although the raw soy sauce was separated from residues via hot extraction, the EC content of soy sauce residue in Cantonese soy sauce was 4.1 μg/kg, accounting for almost two-thirds of each final fermentation broth. In two soy sauces made in the laboratory, EC contents of soy sauce residues whose *moromi* fermented at 15 °C and 30 °C were 3.5 μg/kg and 6.2 μg/kg, respectively. Therefore, filtration with soy sauce residues could be effective EC reduction methods. 

In general, yeast was added to the *moromi* when the pH value was approximately 5 [25], meanwhile, alcoholic fermentation starts to occur. Moreover, when EC was first detected in the three kinds of soy sauce, the pH of all fermentation broths was decreased to 4.8–4.9, suggesting that EC began to accumulate at the stage of alcoholic fermentation. pH showed a significant negative correlation with EC, ethanol and citrulline, but raising broth pH is unadvisable because yeasts could not to survive during brine fermentation when the pH value was higher than 5.0 [25,26]. pH had little effect on EC formation when the pH of raw soy sauce was in the range of 4.6–5 (Figure 3). Moreover, the pH values of raw soy sauce in this study and the majority of soy sauces were in the range of 4.6–5 [14,27]. Therefore, the pH might affect the accumulation of EC precursors during fermentation, but it hardly influences the formation of EC during the heat treatment of soy sauce, unless the pH value of raw soy sauce is artificially adjusted to above 6. Low fermentation temperature not only significantly decreased the final EC content, but also delayed the day when EC was first detected, although the possible EC precursors (urea and citrulline) had accumulated before EC was detected, and their contents in the *moromi* were even higher than those in rice wine [28]. Moreover, a low fermentation temperature was unfavorable for citrulline and ethanol accumulation. In brief, lower initial fermentation temperature and non-thermal method for raw soy sauce extraction can be adopted for EC reduction, in addition, improvement of the pH value of raw soy sauce might reduce the increase of EC after heat treatment process.

## 4. Materials and Methods 

### 4.1. Reagents

EC (99%), urea (99%), 9-xanthydrol (98%), barbituric acid, pyridine and the internal standard isotope labelled ethyl carbamate (EC-d_5_) were purchased from Aladdin (Shanghai, China). Cyanide standard solution (2 mg/L) was purchased from AOKE (Beijing, China). Ethyl acetate, ethanol and diethyl ether were purchased from RCI Labscan Ltd. (Bangkok, Thailand). Sodium hydroxide, acetic acid, sodium sulphate anhydrous and hydrochloric acid were purchased from Heowns (Tianjin, China).

### 4.2. Sample Preparation

**Figure 4:**
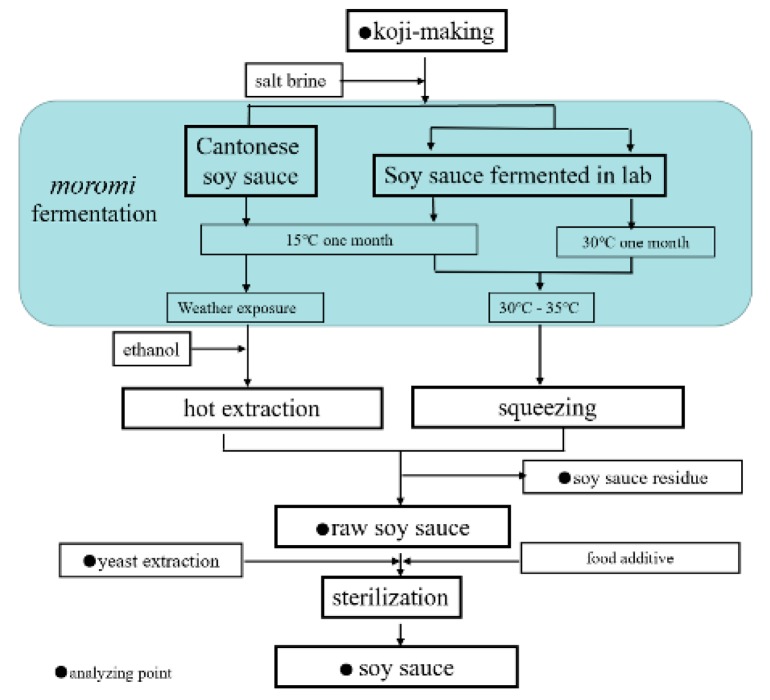
The diagram of soy sauce producing scheme (The arginine, citrulline, and ornithine contents of yeast extraction in this study are 64.71 mg/g, 0 mg/g and 10.89 mg/g, respectively).

The preparation procedures of soy sauce are presented in Figure 4. Cantonese soy sauce was produced by a local Cantonese soy sauce manufactory in Guangdong province, China. The *koji*-making and fermentation procedures were similar to those in previous reports [18,29]. A mixture of steamed soy beans and roasted and grinded wheat (approximate 1:3, *w*/*w*) was incubated with *Aspergillus oryzae* and kept at proper temperature (28–38 °C) and humidity (97–100%), resulting in soy sauce *koji*. The *koji* was mixed with two times volume of salt brine (approximately 18% of salt concentration, *w*/*v*) to yield *moromi*. The *moromi* was incubated at 15 °C for a month and then fermented at natural temperature, and finally resulted in a formation of ripened soy sauce mash. Hot extraction method (soaking at 60–70 °C for 6 h) was adopted for separating raw soy sauce from soy sauce residue. The other two soy sauce samples were fermented in the laboratory with different initial fermenting temperatures. The *koji* used in the laboratory were provided by a Cantonese soy sauce manufactory. The *moromi* contained 1.5 kg *koji*, and two times volume of salt brine was transferred into a 5-L fermentor. The fermentors were randomly divided into two groups, which were separately placed into two thermostatic incubators set at 30 °C and 15 °C in the first month and then allowed continue to ferment at 30–35 °C in the same environment. Raw soy sauce was separated from sauce residue by squeezing on day 90. After pressing, the filtered raw soy sauce was pasteurized at 85 °C for 30 min to inactivate residual enzymes and microorganisms. 

### 4.3. Heat Treatment of Soy Sauce

Aliquots of raw soy sauce (20 mL) with different contents of additives (1% and 2% ethanol, 20 mg/L and 40 mg/L urea, 2 mg/mL and 4 mg/mL citrulline) were transferred into 30 mL sealed cylindrical glass vials. The pH of the mixture was adjusted to the same as that of the original raw soy sauce. Then, the vials were boiled in a water bath at 85 °C for 30 min. Afterwards, these vials were immediately cooled down in an ice bath, and their EC contents were subsequently analyzed by GC-MS. Raw soy sauces with pH values of 6, 5 and 4.6 (adjusted by acetic acid or sodium hydroxide) were heated as described previously.

### 4.4. Determination of EC

The EC content was determined by GC-MS with some modifications [30]. Two grams of sample was spiked with 100 ng internal standard (EC-d_5_), and the mixture was loaded in a solid-phase extraction column (ANPEL Laboratory Technologies Inc. (Shanghai, China)) and kept static for 10 min. After being washed with hexane, the analyte was eluted with 10 mL of ethyl acetate-diethyl ether solution (5% *v*/*v*) at a rate of one drop per second. The eluent was evaporated to 0.5 mL via N_2_ flow at room temperature and brought to a volume of exactly 1 mL for testing. The GC-MS method was carried out by an Agilent 7890A-5975C, (California, USA) with a VF-WAX column (Chicago, IL, USA). The carrier gas was helium at a flow rate of 1.0 mL/min. Sample aliquots of 2.0 μL were injected in splitless mode. The oven temperature program was as follows: 50 °C (1 min), followed by an increase to 180 °C at 8 °C/min and then an increase to 240 °C at 40 °C/min (5 min). The mass spectrometer was operated in electron impact ionization mode at 70 eV. The injector port and mass spectrometer interface line were heated to 220 °C and 250 °C, respectively. The mass spectrometer was operated in scan mode for EC qualification and SIM mode for quantification. The monitored fragment ions had *m*/*z* values of 44, 62, 74 and 89 for EC and *m*/*z* values of 64, 76 and 94 for EC-d_5_. EC was quantified using calibration curves made from peak area ratios of EC/EC-d_5_ (*m*/*z* 62 vs. *m*/*z* 64).

### 4.5. Determination of Urea

The urea content was detected by high-performance liquid chromatographic method coupled with fluorescence detection (HPLC-FID) [31]. Briefly, the sample was diluted 10–20 folds by distilled water. The 0.4 mL diluted sample was mixed with 0.1 mL HCl (1.5 mol/L) and 0.6 mL 9-xanthydrol (0.02 mol/L) and held for 30 min in a dark place before analysis. An excitation wavelength of 213 nm and an emission wavelength of 308 nm was used for the characterization of urea derivative.

### 4.6. Determination of Free Amino Acid

Free amino acids content was determined by a Hitachi automatic amino acids analyzer L-8800 (Honshu, Japan) with the column of LCA K07/Li. Samples were mixed with an equal volume of 6% sulfosalicylic acid for deproteinization (4 °C, 1 h). Precipitate was removed by centrifugation (4 °C, 16,099× *g* for 10 min), and the clear supernatant extract was analyzed. All preparations were diluted appropriately (by sodium citrate buffer, pH 2.2) and filtered with 0.22 μm filter membrane. The amino acid content was determined by Formula 1.

(1) $$C_{x}=\frac{A_{x}}{A_{y}}\times C_{y}\times F$$

$C_{x}$: Amino acid content of analyte sample, $C_{y}$: Amino acid content of standard, F: The sample dilution multiple, $A_{x}$: Peak area of amino acid in analyte sample, $A_{y}$: Peak area of amino acid in standard. $A_{x}$ and $A_{y}$ were obtained from Figure S1.

### 4.7. Determination of Alcohol and Cyanide Content

A 50 g Sample was mixed with 50 mL distilled water in a 500 mL flask, and the mixture was distilled by a distillation unit. The collected distillation (approximate 45 mL) was transferred to a 50 mL volumetric flask and made to volume up to exactly 50 mL with distilled water at 20 °C. Alcoholic content (*v*/*v*) was determined by using potassium bichromate colorimetry [32], and cyanide content was determined using the method of Haskins [33].

### 4.8. Measurement of Metal Content and pH

The determination of metal content was based on flame atomic absorption spectrophotometric method (SpectrAA 220FS/220Z, Palo Alto, CA, USA) [34]. Samples were pretreated by wet digestion, and standard curves were recalibrated for each trail. The sample volume of pure water instead of soy sauce sample was used as blank and digested by using the same method. The pH of soy sauce was given by pH meter (PHS-25, Shanghai, China), and samples were placed at 25 °C for 20 min to guarantee thermal stability before measurements.

### 4.9. Statistical Analysis

Cantonese soy sauce was sampled from three fermented tanks in the same batch. The *moromi* was filtered with through multi-layer gauze before analysis, and each analysis was performed at least in triplicate. The data, including significance and correlation, were analyzed by SPSS Statistics 19, and a statistical significance of 0.05 was used (Tukey HSD). All diagrams were made by Origin 8.

## Figures and Tables

**Table 1:** pH in three kinds of soy sauce during fermentation stage.

Cantonese Soy Sauce		Soy Sauce Fermented at 30 °C		Soy Sauce Fermented at 15 °C	
days	pH	days	pH	days	pH
5	5.87 ± 0.01	5	6.02 ± 0.01	5	6.02 ± 0.01
10	5.20 ± 0.10	10	5.15 ± 0.21	10	5.61 ± 0.14
15	5.29 ± 0.03	20	4.88 ± 0.03	20	5.51 ± 0.11
20	5.24 ± 0.04	30	4.82 ± 0.05	30	5.01 ± 0.06
30	5.17 ± 0.02	45	4.70 ± 0.02	45	4.88 ± 0.06
45	4.88 ± 0.01	60	4.65 ± 0.04	60	4.66 ± 0.03
60	4.85 ± 0.01	90	4.65 ± 0.04	90	4.69 ± 0.04
75	4.89 ± 0.02	Raw soy sauce	4.65 ± 0.02	Raw soy sauce	4.69 ± 0.04
90	4.83 ± 0.01	Soy sauce	4.80 ± 0.02	Soy sauce	4.77 ± 0.02
105	4.81 ± 0.01				
120	4.69 ± 0.02				
135	4.63 ± 0.02				
150	4.48 ± 0.04				
160	4.55 ± 0.01				
Raw soy sauce	4.53 ± 0.02				
Soy sauce	4.72 ± 0.03				

**Table 2:** Mean value of citrulline, ornithine and arginine (μg/mL) in three kinds of soy sauce.

**Cantonese Soy Sauce**							
Days	5	20	60	90	135	165	Soy sauce
citrulline	308 ± 20	574 ± 21	1093 ± 15	1794 ± 112	2234 ± 62	2358 ± 64	1222 ± 62
ornithine	172 ± 10	1045 ± 102	66 ± 42	100 ± 50	612 ± 18	548 ± 12	484 ± 16
arginine	1076 ± 83	1332 ± 138	3725 ± 35	3790 ± 230	3230 ± 270	3475 ± 275	2804 ± 185
**Soy Sauce Fermented at 30 °C**							
Days	5	20	45	90	Soy sauce		
citrulline	163 ± 34	807 ± 46	903 ± 82	1428 ± 27	1054 ± 55		
ornithine	186 ± 20	633 ± 33	74 ± 55	2984 ± 234	1588 ± 84		
arginine	574 ± 138	1610 ± 122	2118 ± 184	1060 ± 46	1020 ± 140		
**Soy Sauce Fermented at 15 °C**							
Days	5	20	45	90	Soy sauce		
citrulline	254 ± 28	518 ± 62	705 ± 55	922 ± 64	768 ± 66		
ornithine	34 ± 12	1800 ± 160	74 ± 22	385 ± 52	392 ± 83		
arginine	718 ± 86	472 ± 106	2052 ± 210	3390 ± 180	2135 ± 185		

**Table 3:** Correlation coefficients between EC, alcohol, cyanide, urea and three kind of free amino acid in each soy sauce.

Soy Sauce Sample	Item	Ethanol	Cyanide	Urea	Citrulline	Ornithine	Arginine	pH
Cantonese soy sauce	EC	0.97 **	0.04	0.26	−0.12	0.07	−0.17	0.70 **
	ethanol		0.08	0.297	−0.10	0.14	−0.19	0.74 **
	cyanide			−0.30	−0.23	0.38	−0.86 *	0.25
	Urea				0.78 *	−0.05	0.15	−0.81 *
	citrulline					−0.03	0.43	−0.97 *
	ornithine						−0.61	−0.09
	arginine							−0.46
Soy sauce fermented at 30 °C	EC	0.79 **	0.52	0.76 *	0.82	0.61	0.20	−0.79 *
	ethanol		0.71 *	0.83 **	0.89 *	0.50	0.65	−0.87 *
	cyanide			0.37	0.49	−0.04	0.80	−0.71
	urea				0.75	0.99 **	−0.36	−0.45
	citrulline					0.80	0.31	−0.90 *
	ornithine						−0.32	−0.45
	arginine							−0.73
Soy sauce fermented at 15 °C	EC	0.69 *	−0.22	0.72 *	0.59	−0.24	0.53	−0.80 *
	ethanol		0.15	0.85 **	0.90 *	−0.38	0.94 *	−0.79 *
	cyanide			−0.03	0.36	−0.15	0.25	−0.37
	urea				0.95 *	−0.36	0.94 *	−0.86 *
	citrulline					−0.06	0.89 *	−0.98 *
	ornithine						−0.44	0.13
	arginine							−0.87

** Correlation is significant at the 0.01 level (2-tailed). * Correlation is significant at the 0.05 level (2-tailed).

## Supplementary Materials

The following are available online at https://www.mdpi.com/1420-3049/24/8/1474/s1, Figure S1: Amino acid chromatogram of standard (a) and soy sauce sample (b), Figure S2: Changes of cyanide in Cantonese soy sauce and soy sauce fermented in the lab, Table S1: Content of Cu, Fe, Ca, Mg in three kinds of fermentation broth, Table S2: Mean value of free amino acid (mg/mL) in three kinds of soy sauces.
