# Supplementary material for: Formation of Ethyl Carbamate during the Production Process of Cantonese Soy Sauce
Source: Molecules. 2019 Apr 15;24(8):1474. doi: 10.3390/molecules24081474 (PMC6514843; doi:10.3390/molecules24081474)
Supplement: Supplementary file 1 [file molecules-24-01474-s001.pdf]

Supporting Information

Formation of Ethyl Carbamate during the Production Process of Cantonese Soy Sauce

Kai Zhou [1], Lorenzo Siroli [2], Francesca Patrignani [2], Yuanming Sun [1], Rosalba Lanciotti [2] and Zhenlin Xu [1,*]

[1] Guangdong Provincial Key Laboratory of Food Quality and Safety, College of Food Science, South China Agricultural University, Guangzhou 510642, China; zkjy1990@163.com (K.Z.); ymsun@scau.edu.cn (Y.S.)

[2] Department of Agricultural and Food Sciences, Alma Mater Studiorum, University of Bologna, 47521 Cesena, Italy; lorenzo.siroli2@unibo.it (L.S.); francesca.patrignani@unibo.it (F.P.); rosalba.lanciotti@unibo.it (R.L.)

* Correspondence: jallent@163.com; Tel.: +86-20-8528-3448; Fax: +86-20-8528-0270

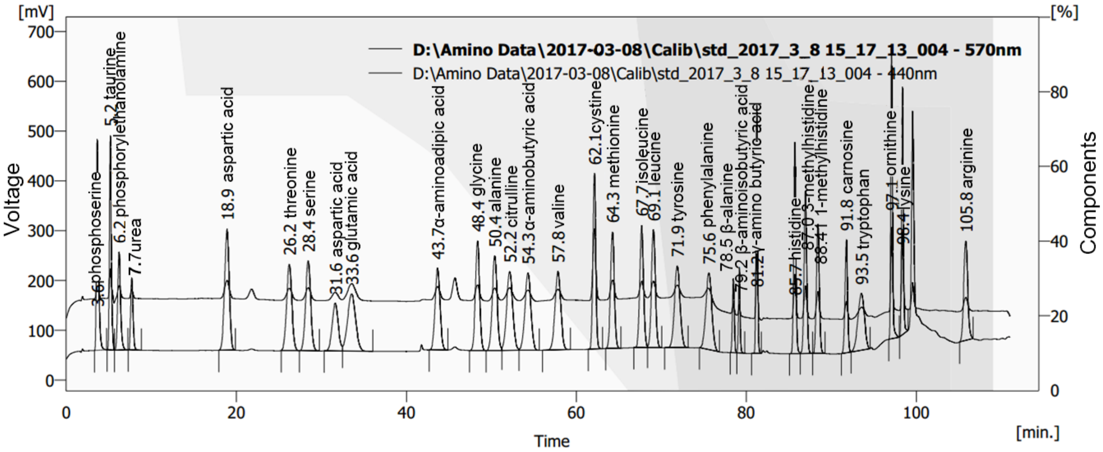

(a)

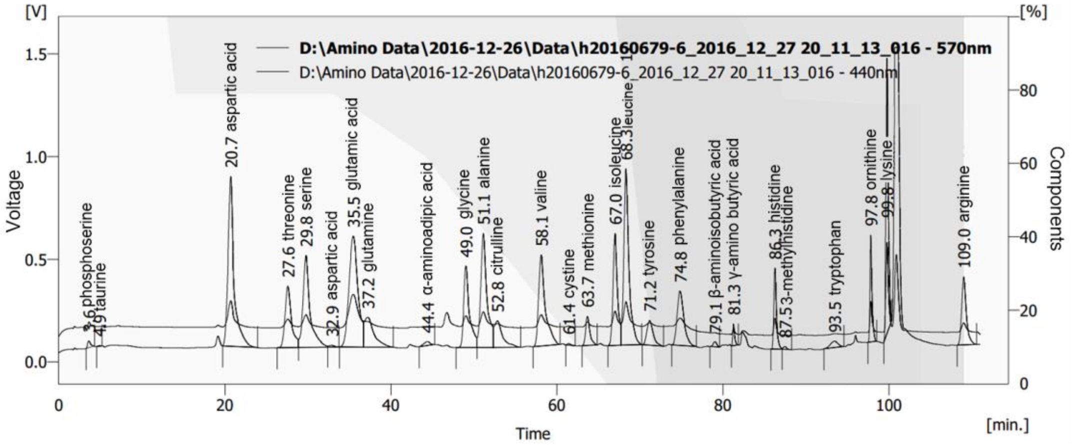

(b)

Figure S1. Amino acid chromatogram of standard (**a**) and soy sauce sample(**b**).

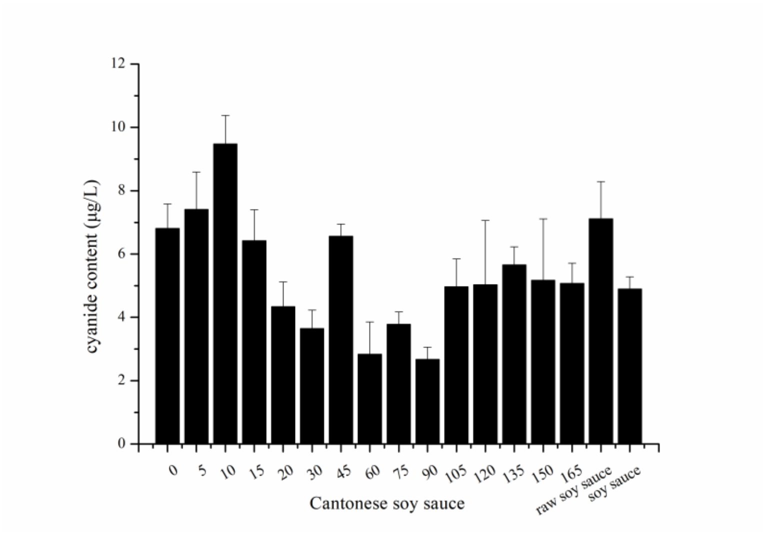

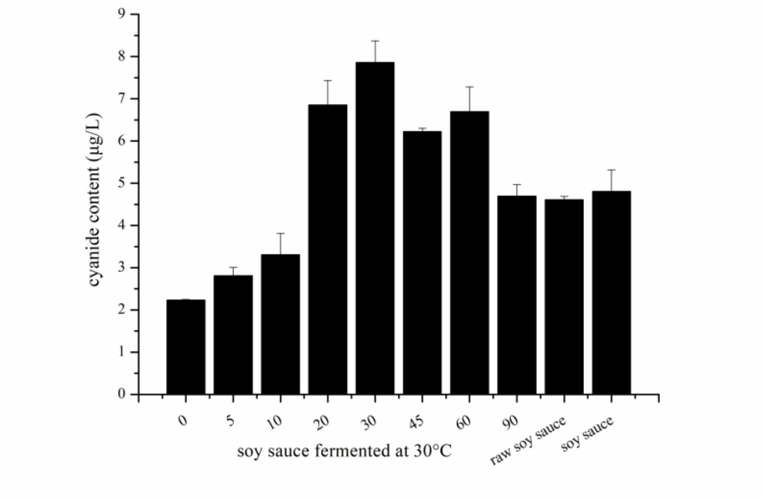

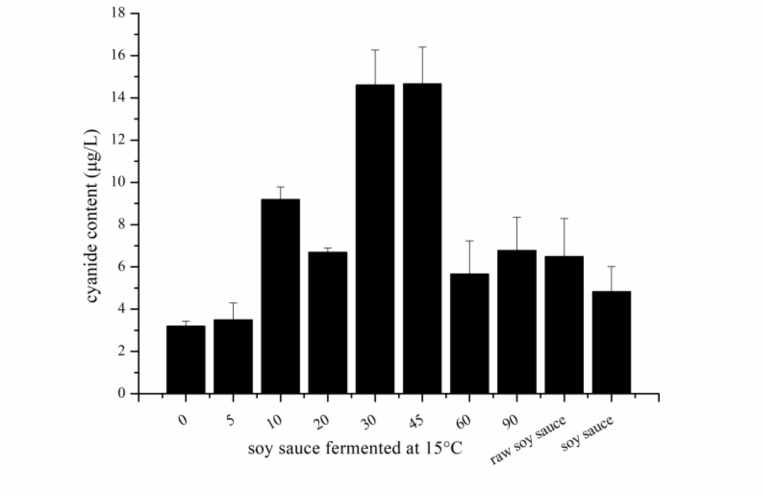

Figure S2. Changes of cyanide in Cantonese soy sauce and soy sauce fermented in the lab.

Table S1. Content of Cu, Fe, Ca, Mg in three kinds of soy sauces.

mg/L	Cu	Fe	Ca	Mg
Cantonese soy sauce	0.79 ± 0.12	50.78 ± 7.26	275.18 ± 10.32	857.33 ± 53.08
Soy sauce fermented at 30°C	1.48 ± 0.16	69.03 ± 6.52	346.65 ± 26.38	925.01 ± 57.09
Soy sauce fermented at 15°C	1.28 ± 0.20	60.21 ± 4.31	348.29 ± 18.33	900.04 ± 18.88

Table S2. Mean value of free amino acid (mg/mL) in three kinds of soy sauces.

mg/mL	Days	Asp	Thr	Ser	Glu	Gly	Ala	Val	Cys	Met	Ile	Leu	Tyr	Phe	His	Lys	Pro
Cantonese soy sauce	5	1.32	0.61	0.68	2.23	0.37	0.71	0.85	0.02	0.14	0.81	1.22	0.44	0.68	0.34	0.17	0.78
	20	2.3	1.07	1.85	6.19	1.14	1.99	1.63	0.03	0.46	1.67	2.65	0.06	1.49	0.3	2.48	1.26
	60	3.33	2.21	3.01	6.67	1.51	2.49	2.48	0.24	0.83	2.6	4.25	1.12	2.35	1.36	4.14	1.26
	90	2.9	1.88	2.61	5.87	1.36	2.19	2.1	0.18	0.71	2.27	3.68	0.82	2.04	1.1	3.53	1.37
	135	3.31	2.07	2.82	6.5	1.47	2.46	2.31	0.19	0.74	2.45	3.88	0.69	2.17	1.16	3.8	1.7
	165	4.1	2.18	3.15	7.87	1.76	2.74	2.7	0.12	0.82	2.8	4.38	0.64	2.45	1.3	4.13	2.06
	Soy sauce	2.81	1.75	2.52	10.08	1.38	3.25	2.41	0.13	0.52	2.35	3.6	0.48	2.66	0.38	2.01	2.71
Soy sauce fermented at 30°C	5	0.85	0.31	0.34	1.08	0.23	0.46	0.54	0.03	0.15	0.52	0.88	0.63	0.74	0.27	0.83	0.2
	20	3.53	1.79	2.33	5.28	1.36	2.28	2.32	0.03	0.63	2.41	3.76	0.78	2.18	0.9	3.07	1.77
	45	2.36	1.38	1.79	5.18	0.93	1.66	1.59	0.19	0.56	1.72	2.85	1.31	1.78	0.89	2.51	1.06
	90	6.08	2.3	2.33	6.58	1.68	3.73	3.83	0.09	1.02	3.53	5.58	1.36	2.92	1.51	5.46	1.91
	Soy sauce	4.16	2.28	2.02	6.49	1.8	3.74	3.4	0.04	0.73	3.15	4.81	0.61	2.65	0.5	2.91	2
Soy sauce fermented at 15°C	5	0.68	0.45	0.58	1.7	0.29	0.57	0.49	0.06	0.19	0.54	0.95	0.48	0.58	0.29	0.83	0.35
	20	3.11	1.43	1.65	5.19	1.08	2.15	1.97	0.03	0.56	2.09	3.18	0.75	1.92	0.93	3.18	1.2
	45	1.96	1.25	1.6	4.62	0.81	1.55	1.38	0.17	0.52	1.48	2.59	1.27	1.64	0.8	2.3	0.98
	90	3.13	2	2.38	5.44	1.34	2.9	2.97	0.16	0.89	2.78	2.78	1.81	2.04	1.47	4.56	1.44
	Soy sauce	2.19	2.07	3.81	5.84	1.35	3.08	2.93	0.1	0.89	3.62	2.62	0.75	3.08	0.77	3.55	1.77
